# Cytomegalovirus-specific T-cell responses and viral replication in kidney transplant recipients

**DOI:** 10.1186/1479-5876-6-29

**Published:** 2008-06-09

**Authors:** Adrian Egli, Isabelle Binet, Simone Binggeli, Clemens Jäger, Alexis Dumoulin, Stefan Schaub, Juerg Steiger, Urban Sester, Martina Sester, Hans H Hirsch

**Affiliations:** 1 Transplantation Virology, Institute for Medical Microbiology, University of Basel, Petersplatz 10, 4003 Basel, Switzerland; 2 Nephrology, Kantonsspital St. Gallen, Switzerland; 3 Nephrology and Transplantation Immunology, University Hospital of Basel, Petersgraben 4, 4031 Basel, Switzerland; 4 Medical Department IV, Nephrology, and Department of Virology, University of Saarland, Homburg, Germany; 5 Infectious Diseases and Hospital Epidemiology, University Hospital of Basel, Petersgraben 4, 4031 Basel, Switzerland

## Abstract

**Background:**

Cytomegalovirus (CMV) seronegative recipients (R-) of kidney transplants (KT) from seropositive donors (D+) are at higher risk for CMV replication and ganciclovir(GCV)-resistance than CMV R(+). We hypothesized that low CMV-specific T-cell responses are associated with increased risk of CMV replication in R(+)-patients with D(+) or D(-) donors.

**Methods:**

We prospectively evaluated 73 consecutive KT-patients [48 R(+), 25 D(+)R(-)] undergoing routine testing for CMV replication as part of a preemptive strategy. We compared CMV-specific interferon-γ (IFN-γ) responses of CD4+CD3+ lymphocytes in peripheral blood mononuclear cells (PBMC) using three different antigen preparation (CMV-lysate, pp72- and pp65-overlapping peptide pools) using intracellular cytokine staining and flow cytometry.

**Results:**

Median CD4+ and CD8+T-cell responses to CMV-lysate, pp72- and pp65-overlapping peptide pools were lower in D(+)R(-) than in R(+)patients or in non-immunosuppressed donors. Comparing subpopulations we found that CMV-lysate favored CD4+- over CD8+-responses, whereas the reverse was observed for pp72, while pp65-CD4+- and -CD8+-responses were similar. Concurrent CMV replication in R(+)-patients was associated with significantly lower T-cell responses (pp65 median CD4+ 0.00% vs. 0.03%, p = 0.001; CD8+ 0.01% vs. 0.03%; p = 0.033). Receiver operated curve analysis associated CMV-pp65 CD4+ responses of > 0.03% in R(+)-patients with absence of concurrent (p = 0.003) and future CMV replication in the following 8 weeks (p = 0.036). GCV-resistant CMV replication occurred in 3 R(+)-patients (6.3%) with pp65- CD4+ frequencies < 0.03% (p = 0.041).

**Conclusion:**

The data suggest that pp65-specific CD4+ T-cells might be useful to identify R(+)-patients at increased risk of CMV replication. Provided further corroborating evidence, CMV-pp65 CD4+ responses above 0.03% in PBMCs of KT patients under stable immunosuppression are associated with lower risk of concurrent and future CMV replication during the following 8 weeks.

## Background

Potent immunosuppressive drug regimens have led to a significant decline of acute and chronic immune reactions in solid organ transplantation (SOT) with increased graft survival across HLA mismatches [1,2]. However, complications associated with impaired immunity have become more prevalent [3,4]. Cytomegalovirus (CMV) is notorious for exerting direct and indirect effects affecting graft and patient survival, despite the availability of validated strategies for prophylactic, preemptive and therapeutic intervention [5-7]. Persistent CMV replication has been linked to poor graft outcomes, even in the absence of classical signs of disease [8-10]. The risk of CMV replication and disease after SOT is higher in seronegative recipients R(-) of seropositive donor D(+) organs than in seropositive R(+) recipients [11] suggesting that CMV-specific immunity provides a certain degree of protection despite maintenance immunosuppression. Prophylaxis with oral ganciclovir (GCV) or valganciclovir (valGCV) has been recommended for D(+)R(-) high-risk patients [12,13]. However, occurrence of GCV-resistance has been reported [14,15]. Cytotoxic CD8+ T-cells are thought to play a major role in terminating CMV replication, while CMV-specific CD4+ T-cells have been linked to long-term antiviral control [16,17]. A better understanding of CMV-specific T-cell immunity in transplant patients is therefore of high interest, particularly in the preemptive setting when prophylaxis is not used. Different CMV antigens and read-out assays yielded seemingly contradictory results in SOT recipients [17-24]. Tetramer-based protocols are very sensitive to identify and characterize CMV-specific CD4+ and CD8+T-cell populations, but restriction to single HLA antigens and knowledge of epitopes prohibits widespread application in the clinical routine [25]. This limitation may be overcome by stimulating T-cells with lysates from CMV-infected fibroblast or by using synthetic overlapping 15 mer peptide pools covering dominant viral proteins such as CMV pp65 or pp72 [26]. Flow-cytometry and Elispot assays detecting Interferon-γ (IFNγ) expression have been used to identify CMV-specific CD4+ and CD8+ T-cells in recipients of liver [19,20], heart and lung [17,21] and kidney transplant (KT) [21-24]. Sester found that increasing calcineurin inhibitor concentrations correlated with impaired IFNγ-responses to CMV-lysate, and correspondingly lower responses in heart and lung than in KT patients [23]. Bunde and colleagues reported that pp72- but not pp65-specific CD8+-responses correlated with protection from CMV disease, but not from CMV replication in heart and lung transplant patients [17]. However, a recent study of 20 D(+)R(-) liver transplants could not correlate either pp72- or pp65- responses with protection from CMV disease [19]. By contrast, Lilleri et al. [21] found that protection from CMV replication of 16 R(+) SOT recipients (heart, lung, kidney) correlated with strong T-cell responses when antigens were presented by CMV-infected autologous dendritic cells. For R(+) KT patients, Radha et al. [22] demonstrated that pp65-specific CD4+ T-cell responses was associated with rapid CMV clearance which was also observed for D(+)R(-) patients developing high CD8+ T-cell responses. We hypothesized that CMV-seropositive D(+)R(+) and D(-)R(+) KT patients with low CMV-specific T-cell frequencies are at increased risk for CMV replication. In view of the controversial information, we decided to re-assess the association of CMV-specific immune responses and CMV replication in the clinical routine setting and enrolled in 73 consecutive KT patients undergoing routine testing for CMV replication as part of the preemptive management followed in our centers. In patients with persistent CMV replication, we searched for mutations conferring GCV-resistance in the CMV UL97 gene.

## Patients and methods

### Patient population

**Figure 1:**
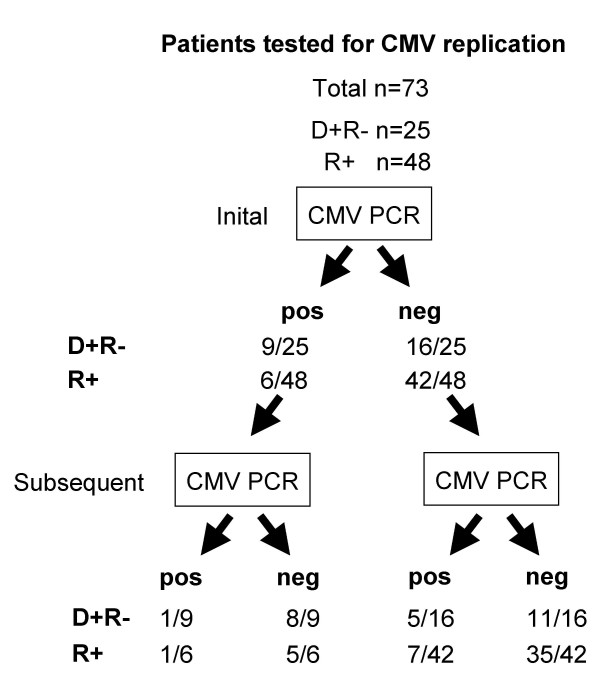
**Study flow chart**. We enrolled patients undergoing testing for CMV replication as part of the preemptive management strategy. CMV-PCR testing was performed together with the measurement of CMV-specific T-cell responses. According to the initial CMV PCR results, the patients were divided into the 2 groups of CMV replication positive and negative. Further viral testing was done and patients were then newly assigned to a CMV PCR positive or negative. The corresponding numbers of D(+)R(-) and R(+) are shown.

Consecutive adult CMV D(+)R(-), D(+)R(+) or D(-)R(+) KT patients (n = 73) were enrolled in this prospective cross-sectional study. Participants were enrolled in Basel and in St. Gallen according to the protocol approved by the internal review board (299/06) in compliance with the declaration of Helsinki. Patients were entered into the study if blood was monitored for CMV replication as part of a preemptive strategy (Figure 1). No prophylaxis was administered, but testing of CMV D(+)R(-), and D(+)R(+) or D(-)R(+), thereafter referred to as R(+) KT patients was performed bi-weekly for the first 4 months, then monthly until months 6. Cases with documented CMV replication were tested weekly until resolution was confirmed (Table 1). D(-)R(-) patients were considered low risk and not routinely tested. All blood samples including the PBMCs were taken before intake of daily drugs. Between antiviral or immunosuppressive drugs and blood sampling were at least 12 h. Patients were treated with GCV or ValGCV when CMV replication was documented according to Preiksaitis et al [12], Sanford Guidelines and renal function. For values close to the diagnostic cut-off of 1000 cp/mL, confirmatory testing was done within 1 week. At the time of sampling, antivirals were administered to 10/25 D(+)R(-) (val-GCV in 9, intravenous GCV in 1; concurrent CMV replication in 4 cases) and to 12/48 R+ patients (valganciclovir in 12; concurrent CMV replication in 6 cases). Intravenous GCV was used for in-patients, out-patients were treated with ValGCV. Oral GCV was never used. 71/73 patients received de-novo transplantation. 2/73 patients were re-transplanted, but did not develop CMV replication. At the time of laboratory testing for CMV replication and cellular immune responses, triple immunosuppression was administered in 42 patients (58%; CMV replication in 7 cases), dual immunosuppression in 24 cases (33.3%, CMV replication in 5 cases) and monotherapy was used in 2 cases, 1 with CMV replication. Tacrolimus was used in 51 patients (71%) with mean trough levels 8.71 ng/mL, 13 showing CMV viremia (p = 0.038, compared to not tacrolimus-treated patients; chi-squared). Mycophenolate mofetil was used in 41 patients (57%) with mean trough levels of 2.54 ug/mL, 6 showing CMV viremia (p = 0.311; chi-squared). Sirolimus was used in 18 patients (25%; mean trough levels 6.92 ng/mL, no CMV replication), cyclosporine A in 10 patients (14%; mean trough levels 288 ng/mL, no CMV replication); azathioprine in 14 patients (19%, CMV replication in 3 cases); leflunomide in 3 patients (4.2%; CMV replication in 3 patients); prednisone in 41 patients (57%; mean dosing 9.3 mg/day; CMV replication in 7 cases). We also tested 30 non-immunosuppressed healthy donors (HD) [17 seropositive HD(+): median age 31 (range 22–48), 8 males; 13 seronegative; HD(-): median age 32 (range 27–60), 9 males].

**Table 1:** Characteristics of CMV replicating and non-replicating kidney transplant patients.

	**CMV D+R-**			**CMV D+R+/D-R+**		
	
Initial CMV replication	all	yes	no	all	yes	no
number^a^	25	9	16	48	6	42
Age, median years (range)	60 (18–71)	63 (25–71)	59 (18–71)	49 (21–73)	53 (43–70)	43 (21–73)
Gender (m/f)	15/10	6/3	9/7	33/15	5/1	28/14
Sample date, weeks postTx median (range)	18 (2–383)	30 (6–41)	15 (2–383)	27 (2–314)	21 (5–138)	17 (2–314)
Induction (%)	19 (76.0%)	7 (77.8%)	12 (54.5%)	36 (75.0%)	3 (50.0%)	33 (76.7%)
T-cell depleting induction (%)	1 (4.0%)	0 (0.0%)	1 (4.5%)	4 (8.3%)	0 (0.0%)	4 (9.3%)
AR therapy (%)	8 (32.0%)	2 (22.2%)	6 (27.3%)	21 (43.8%)	3 (50.0%)	18 (41.9%)
T-cell depleting AR therapy (%)	3 (12.0%)	0 (0.0%)	3 (13.6%)	11 (22.9%)	2 (33.3%)	9 (20.9%)
Initial CMV load, mean (c/ml)^a^	-	109'900	< 300	-	49'588	< 300
Initial CMV peak, mean (c/ml)	-	181'811	< 300	-	90'009	< 300
Later CMV replication (n/total)^b^	-	1/9	5/16	-	1/6	7/42
Later CMV load, mean (c/ml)	-	288'001	9'323	-	1'489	72'581
Later CMV peak, mean (c/ml)	-	1'240'000	37'260	-	3'387	311'071

Note. Cytomegalovirus (CMV) seronegative KT patients had a D+R- serostatus. CMV seropositive KT patients were D+R+ or D-R+. AR, acute rejection; ATG, anti-thymoglobuline.

^a ^Patients are grouped according to CMV replication at initial time-point of testing PCR and T-cell responses and denoted as initial CMV episode.

^b ^Positive PCR testing detected during follow-up was denoted as later CMV episode.

### CMV diagnostic assays

IgG CMV-serology (AxSym™ assay, Abbott Diagnostics, Baar, Switzerland) was used to identify CMV seropositive and seronegative individuals. CMV replication was quantified after DNA extraction from EDTA-anticoagulated whole blood (MagNApure™, Roche-Diagnostics, Rotkreuz, Switzerland). CMV replication was quantified by real-time-PCR using the primers TTT TTT CTA GGC GCT TCC GA and ACA CTG CGG CTT TGT ATT CTT TAT C, and the FAM-TAMRA labeled probe AGG CGA AGC CGG CGA CGA (Applied Biosystems, Rotkreuz, Switzerland). The linear range of the assay is validated from 10e2 to 10e8 copies (cp)/mL. The limit of detection is 300 cp/mL, and 1000 cp/mL were used as routine diagnostic cut-off value. All assays were performed in triplicates. For quantification, a standard curve was constructed from defined copy number of the cloned targets using 10e6, 10e4, 10e2 cp contained in 5 uL which was added to each reaction. Since DNA was extracted from 200 μL blood and eluted into 100 μL (e.g. 2-fold concentration), 5 μL per assay correspond to 10 μL extracted blood which needed to be multiplied by 100 to obtain the copy number per mL blood i.e. 10e8/mL, 10e6/mL, and 10e4/mL. DNA specimens or controls were added as 5 uL to 20 uL containing 300 nM of the respective primer pair, 200 nM of the respective probe and 12.5 uL of the 2-fold concentrated commercially obtained using a mastermix containing the AmpliTaq polymerase, dNTP mix with dUTP replacing dTTP, Uracyl-N-gylcosylase (Eurogentec, Seraing, Belgium) to yield final volume of 25 μL. Each DNA sample was also analyzed after spiking with 1000 copies of the target pCMV1 to monitor for inhibition. The temperature profile consisted of preincubation at 50°C, 2 min to allow for enzymatic decontamination of synthetic uracyl-containg amplicons, followed by 95°C; 15 min for hot-start activation and 45 cycles of 95°C; 15 sec; 60°C; 60 sec, followed by 7 min at 72°C. In case of inhibition or unclear results, the DNA was extracted once again and assayed as described. Each PCR assay contained routinely non-template controls in triplicates as well as one contamination control of human blood donor serum which was taken through the entire process of DNA extraction and assayed in triplicates.

Clinical GCV-resistance was defined as persistent CMV replication despite adequate antiviral treatment for > 7 days or as breakthrough replication during prophylaxis [12]. Genotypic resistance was diagnosed when known mutations in the CMV UL97 gene were identified using cycle sequencing after a nested PCR strategy with TGC TGC ACA ACG TCA CGG TAC ATC and AAA CAG ACT GAG GGG GCT ACT as outer primers (10 min at 95°C; then 40 cycles of 30 sec at 95°C, 30 sec at 50°C, 1 minute at 72°C and 7 minutes extension at 72°C) followed by amplification of two fragments with CGT TGG CCG ACG CTA TCA AAT TTC and ACA GCT CCG ACA TGC AAT AAC G (348 bp), as well as GTG GGT AAC GTG CTG GGC TTT TG and GTG GGT TTG TAC CTT CTC TGT TGC (518 bp). (10 min at 95°C; then 40 cycles of 30 sec at 95°C, 30 sec at 50°C, 1 minute at 72°C and 7 minutes extension at 72°C). Final concentrations in a 50 μL reaction volume were 1 uM primer, 200 nM dNTP, 1× Pwo buffer, 1U of Pwo polymerase (Roche, Roche-Diagnostics, Rotkreuz, Switzerland). The respective amplicons were isolated from preparative gel electrophoresis for cycle sequencing. If UL97 sequences indicated multiple CMV mutants, the amplicons were cloned in pGEM3Zf+ plasmid (Promega, Wallisellen, Switzerland), and clones were sequenced.

### Quantification of CMV specific T cells

Cellular immune responses were tested in the laboratory without knowledge of the serostatus of donor or recipient. The results of each patient were determined as the mean of duplicate testing in a single blood draw. The number of sampling measurements exceeded the number of patients because some patients were sampled and tested more than once during the observation period. The frequency of CMV-antigen-specific IFNγ-producing T-cells by intracellular cytokine staining was carried out according to a previously published protocol [16] except that peripheral blood mononuclear cells (PBMC) instead of whole blood were used. PBMC were taken before all medication, especially antiviral treatment and immunosuppressive drugs and tested for CD69+ IFNγ+ response in CD4+CD3+ and CD8+CD3+ T-cells after stimulation with three different CMV-antigens: 1) Lysate preparations from CMV infected fibroblast cell cultures (4 ug/ml, Virion, Rüschlikon, Switzerland), 2) peptide pool covering the immediate early protein 1 (pp72), and 3) peptide pool covering the late gene tegument protein (pp65). The peptide pools consisted of 15 amino acids (aa) long peptides with 11aa overlaps and were used in a final concentration of 1 ug/ml (Eurogentec). Added non-infected fibroblast-lysate preparations served as negative control for CMV-lysate and RPMI1640 media alone for peptides. Staphylococcal enterotoxin B (SEB, 1 ug/ml, Sigma-Aldrich, Buchs, Switzerland) served as positive control.

PBMC were recovered either from citrate anti-coagulated CPT™-vacutainers (Becton Dickinson, Allschwil, Switzerland) or from EDTA blood using Lymphprep™ (Axis Shield, Dundee, Scottland), without notable differences in the CMV-specific responses or in the unstimulated control. PBMC were washed twice in phosphate-buffered saline (PBS) and stimulated with CMV-antigens in presence of α CD28/α CD49d (1 ug/ml, Becton Dickinson), tested in serial dilution, data not shown) for total 6 hours in RPMI 1640 medium (Sigma) with 10% fetal calf serum, 1% penicillin/streptomycin (Gibco, Invitrogen, Basel Switzerland) and 1% glutamax (Gibco). After two hours we added brefeldin A (10 ug/ml, Sigma) to prevent IFNγ secretion. Cells were washed once with PBS (without Ca^2+ ^and Mg^2+^, pH 7.2), fixed first with 4%, then with 1% paraformaldehyde, permeabilized with 0.1% saponin (Sigma) in PBS and stained at room temperature in the dark with following antibodies: α CD3, α CD4, α CD8, α CD69 and α IFNγ (all Becton-Dickinson). At least 30'000 CD3+ lymphocytes were analyzed on a FACSCanto (Becton-Dickinson). The frequency of CMV-specific T-cells was analyzed for each antigen and was expressed as percentage of CD69 and IFNγ double positive CD4+CD3+ or CD8+CD3+ cells. Negative controls were subtracted to determine the antigen-specific frequency.

### Data analysis

Data were summarized as mean ± standard deviations (± SD) or as median and ranges where appropriate. When Kolmogorov-Smirnov Z Test indicated lack of normal distribution, nonparametric tests were used such as the Mann-Whitney U test, Spearman's rho correlation analysis, and paired Wilcoxon test. Categorical markers were analyzed by Fisher's exact or Pearson's chi-square test. Binary logistic regression's default and receiver operator characteristics (ROC) analysis with Youlden's Test was used to determine cut-off levels of T-cell responses. Two-sided p-values < 0.05 were considered as statistically significant. Bonferroni correction was in multiple test situations to avoid false level of significance. For statistical analysis, we used the SPSS 13^th ^version package (SPSS, Zurich, Switzerland).

## Results

### Immunosuppression and CMV replication at the time of sampling

Seventy-three KT patients undergoing routine testing for CMV replication were enrolled in the study consisting of 48 D(+)R(+) or D(-)R(+), thereafter referred to as R(+) KT patients and 25 D(+)R(-) KT patients (Table 1). At the time of sampling, triple immuno-suppression was administered in 58%, dual immunosuppression in 33.3%, and monotherapy was used in 2 cases. CMV replication was found in 9 of 25 D(+)R(-) KT patients at the time of the initial CMV immune response test. Subsequent CMV replication was found in another 5 D(+)R(-) patients during the follow-up (Figure 1). Six of 48 R(+) KT patients had CMV replication (reactivation) at the time of initial testing, and subsequent CMV replication was documented in another 7 R(+) KT patients during the follow-up (Figure 1). CMV replication was more frequent in D(+)/R(-) patients than in R(+) patients (p = 0.031 Fisher's exact).

### CMV-specific cellular immune response in kidney transplanted patients

**Figure 2:**
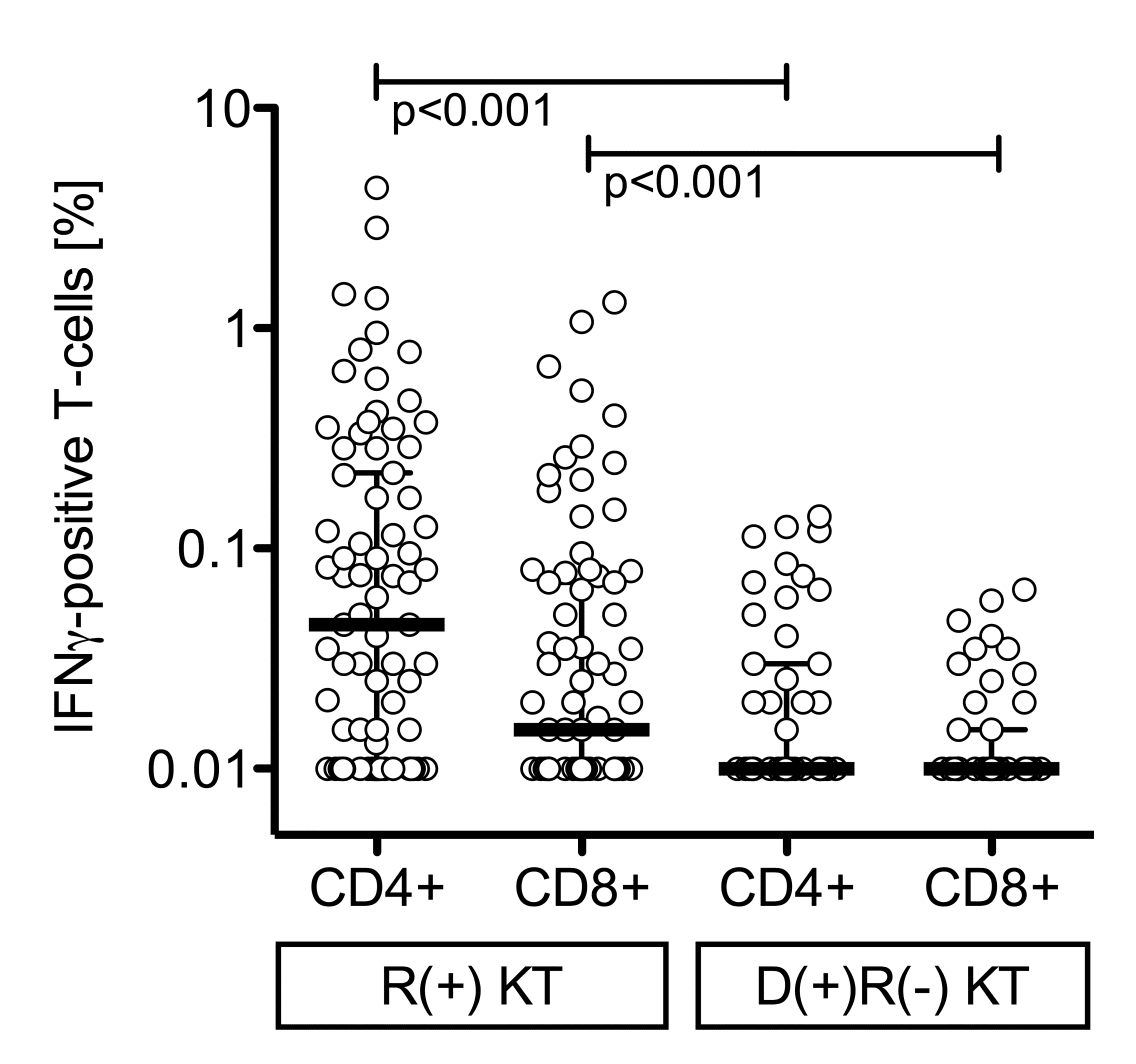
**CMV-specific IFNγ positive T-cell response in KT patients**. CMV-lysate-specific interferon-γ responses in CMV-seropositive R(+) patients and CMV-seronegative D(+)R(-) KT patients. Black bars indicate median, whiskers indicate interquartile range. P-values were calculated using Mann-Whitney U test. Note: The median was below the analytical cut-off of 0.01% in the CMV-seronegative D(+)R(-) KT patients.

PBMC were stimulated with CMV antigens and the frequency of IFNγ-producing T-cell subsets was recorded (Table 2). As shown for CMV-lysate (Figure 2), R(+) KT patients had higher CD4+ and CD8+ T-cell responses than D(+)R(-) KT patients. This was also observed for CMV pp65, but the significance level was barely missed for pp72 stimulated CD8+ T-cells (p = 0.056; Table 2). Non-immunosuppressed HD(+) had higher CMV-specific IFNγ T-cell frequencies than R(+) KT patients for CMV-lysate and pp65 antigens (p < 0.001; Figure 2 and data not shown), except for pp72 for which a strong trend was observed (p = 0.055).

**Table 2:** Percentage of CMV-antigen specific interferon gamma (IFN-γ) producing CD4+ and CD8+ T-cells in healthy donors and kidney transplant patients.

		**Kidney transplant (KT) patients**				**Healthy donors (HD)**		
			**CMV D+R+/D-R+ ** **n = 48, m = 79**	**CMV D+R- ** **n = 25, m = 50**	p-values*	**CMV seropositive ** **n = 13, m = 19**	**CMV seronegative ** **n = 17, m = 17**	p-values*

**CMV-**	CD3^+ ^CD4^+^	median (range)	0.05 (0.00–4.35)	0.01 (0.00–0.14)	< 0.001	1.03 (0.03–6.19)	0.01 (0.00–0.22)	< 0.001
**lysate**	CD3^+ ^CD8^+^	median (range)	0.02 (0.00–1.31)	0.00 (0.00–0.07)	0.003	0.49 (0.00–4.57)	0.00 (0.00–0.56)	< 0.001
**pp72**	CD3^+ ^CD4^+^	median (range)	0.04 (0.00–0.36)	0.02 (0.00–0.26)	0.027	0.08 (0.01–2.83)	0.00 (0.00–0.03)	0.001
**peptides**	CD3^+ ^CD8^+^	median (range)	0.05 (0.00–0.68)	0.03 (0.00–0.34)	0.056	0.2 (0.00–3.47)	0.00 (0.00–0.03)	0.002
**pp65**	CD3^+ ^CD4^+^	median (range)	0.02 (0.00–0.50)	0.01 (0.00–0.55)	0.013	0.11 (0.00–4.70)	0.00 (0.00–0.02)	0.018
**peptides**	CD3^+ ^CD8^+^	median (range)	0.02 (0.00–0.62)	0.00 (0.00–0.25)	0.001	0.21 (0.00–2.80)	0.00 (0.00–0.20)	0.009

Note. The results are given as frequencies of CD3^+ ^CD4+ or CD3+CD8+ lymphocytes as detailed in Materials&Methods. The number of measurements exceeds the number of patients because some patients were tested at more than one visit. CMV, Cytomegalovirus; HD, healthy donors; KT, kidney transplant; n, number of patients; m, number of independent blood sampling and measurements. CMV seronegative KT patients had a D(+)R(-) constellation. CMV seropositive KT patients included both D(+)R(+) and D(-)R(+) constellations. (*) Mann-Whitney U test.

We compared the responses to different CMV-antigens in R(+) KT patients. CMV-lysate specific CD4+ and CD8+ T-cell responses were correlated with pp65-specific CD4+ and CD8+ T-cell responses (Spearman's rho 0.564, 2-tailed p < 0.001; and Spearman's rho 0.514, 2-tailed p < 0.001, respectively). No correlation was found between pp65- and pp72-specific CD4+ T-cells (Spearman's rho 0.133, 2-tailed p = 0.347), or between CMV-lysate specific and CMV-pp72-specific CD8+ T-cell or CD4+ T-cell frequencies (Spearman's rho 0.091, 2-tailed p = 0.530; and Spearman's rho -0.263, 2-tailed p = 0.065, respectively). We concluded that T-cell responses to CMV-lysate and -pp65 appeared similar in PBMC of KT patients and differed from those to pp72.

### CMV-specific cellular immune responses in seropositive KT patients with CMV replication

**Figure 3:**
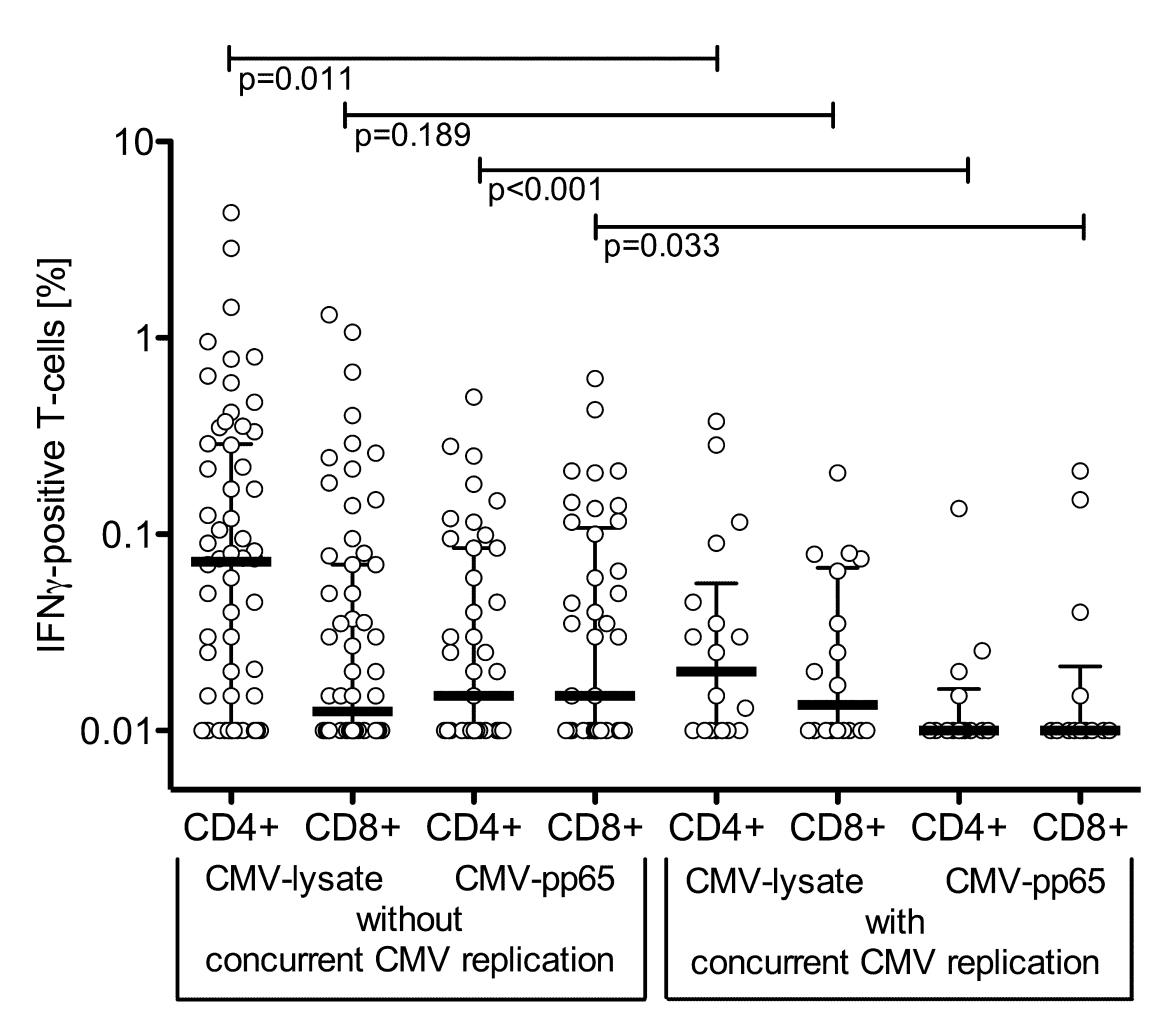
**CMV-specific IFNγ positive T-cell response in KT patients**. CMV-lysate and CMV-pp65 peptide-induced interferon-γ responses in R(+) KT patients with and without concurrent CMV replication. Note: The median was below the analytical cut-off of 0.01% in for the pp65-induced CD 4+ and CD8+ responses in patients with concurrent CMV replication.

To investigate associations with viral control, we compared the frequency of CMV-specific IFNγ responses in R(+) KT patients with and without concurrent CMV replication. Patients with concurrent CMV replication had on average lower CMV-specific CD4+ and CD8+ T-cell responses compared to patients without concurrent CMV replication (Figure 3; see also Table 3). The differences were statistically significant for CD8+ as well as for CD4+ T-cell responses to CMV-pp65 peptide antigens. Significant differences were also found for CD4+ T-cell responses to CMV-lysate and for CD8+ T-cell responses to pp72-peptides (Table 3). However, the complementing CD8+ T-cell responses to CMV-lysate and CD4+ T-cell responses to pp72 were not significantly different. As indicated above, CD8+ T-cell responses to CMV-lysate and CD4+ T-cell responses to pp72 were generally lower suggesting the possibility of a weaker resolution. We concluded that overall the data pointed to an inverse relation of CMV-specific T-cell frequencies and viral replication, which seemed to be best resolved by the pp65-specific CD4+ T-cell responses.

**Table 3:** Percentage of CMV-antigen specific interferon gamma producing CD4+ and CD8+ T-cells of R+ patients with or without concurrent CMV replication.

			**CMV replicating** ** (n = 6/48)**	**CMV non-replicating ** **(n = 42/48)**	p-values*
**CMV-**	CD3+CD4+	median (range)	0.02 (0.00–0.38)	0.08 (0.00–4.35)	0.011
**lysate (m = 79)**	CD3+CD8+	median (range)	0.01 (0.00–0.21)	0.02 (0.00–1.31)	0.189
**pp72**	CD3+CD4+	median (range)	0.04 (0.00–0.22)	0.04 (0.00–0.36)	0.291
**peptides (m = 50)**	CD3+CD8+	median (range)	0.03 (0.00–0.24)	0.07 (0.00–0.68)	0.019
**pp65**	CD3+CD4+	median (range)	0.00 (0.00–0.14)	0.03 (0.00–0.50)	< 0.001
**peptides**	CD3+CD8+	median (range)	0.01 (0.00–0.21)	0.03 (0.00–0.62)	0.033

Note. The results are given as frequencies of CD3^+ ^CD4+ or CD3+CD8+ lymphocytes as detailed in Materials&Methods. The number of measurements exceeds the number of patients because some patients were tested at more than one visit. CMV, cytomegalovirus; R, recipient; n, number of patients; m, number of measurements. CMV seropositive KT patients included both D+R+ and D-R+ constellations. Mann-Whitney U test was used to calculate differences (*)

**Figure 4:**
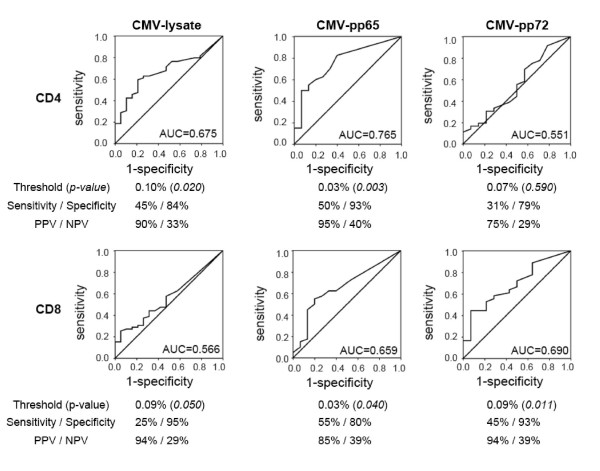
**Receiver operating characteristic (ROC) analysis – CMV-specific T-cells protecting from concurrent CMV replication **ROC analysis shows thresholds of protection from concurrent CMV replication for the different CMV antigens tested. AUC, Area under the curve; p-value by Fisher exact test; PPV, positive predictive value; NPV, negative predictive value.

To identify a possible cut-off of IFNγ T-cell frequency associated with patients being free from concurrent CMV replication, we subjected the data to ROC analyses (Figure 4). For CMV-lysate, absence of concurrent CMV replication was significantly associated with CD4+ T-cell frequencies of > 0.1% and CD8+ T-cell frequencies of > 0.09%. For CMV-pp65, significant threshold values for CD4+ and CD8+ T-cells were both > 0.03%. For CMV-pp72, the cut-offs for CD4+T-cells was > 0.07% (not significant) and for CD8+T-cells was > 0.09% (p = 0.011), respectively. As indicated by the higher area under the curve (AUC) of 0.765, CMV pp65-specific responses > 0.03% seemed to provide the best discrimination for CD4+ T-cell subsets with a positive predictive value for CMV pp65 CD4 > 0.03% of 95% and a negative predictive value of 40% (Figure 4). For CMV-specific CD8 responses, pp72 provided the highest AUC of 0.690, but CD4 pp65 AUC was only slightly lower with 0.659 (positive and negative predictive value of 85% and 39%, respectively).

**Figure 5:**
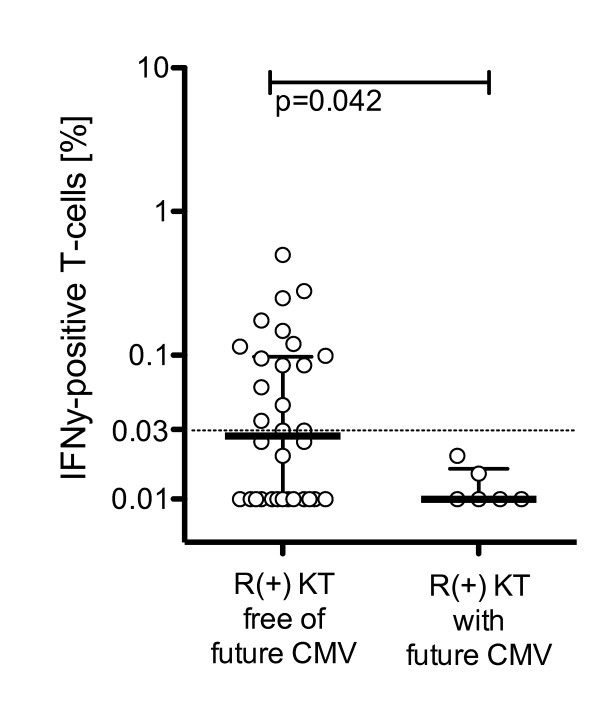
**CMV pp65-specific CD4+ T-cell responses and CMV replication in the following 8 weeks**. X-axis indicates KT R(+) patients without CMV replication in the following 8 weeks versus KT R(+) patients with CMV replication in the following 8 weeks. Y-axis shows frequency of IFNγ positive T-cells in % after specific stimulation with CMV-pp65 peptides. Black bars indicate median, whiskers indicate interquartile range. P-values by Mann-Whitney U test.

To investigate the degree of protection for later CMV episodes, we examined the occurrence of CMV viremia during the follow-up period. Seven of 42 R(+) patients without CMV replication at the initial time-point of measurement had subsequent CMV replication within 8 weeks (median, range 6 to 56 weeks). Here, pp65-specific CD4+ T-cell frequencies were significantly lower compared to R(+) patients without later CMV replication (Figure 5; p = 0.042; Mann-Whitney U test). ROC analysis confirmed that pp65-specific CD4+ T-cell frequencies of > 0.03% were associated with being free from later CMV replication over the following 8 weeks (AUC: 0.763, specificity: 100%, sensitivity: 47%, Fisher exact test: p = 0.036, positive predictive value 100%, negative predictive value 27%). We could not identify a similar cut-off for pp65-specific CD8+ T-cell responses or for any of the CMV-lysate or -pp72 responses (data not shown).

### Ganciclovir-resistance and CMV-specific cellular immune responses

**Figure 6:**
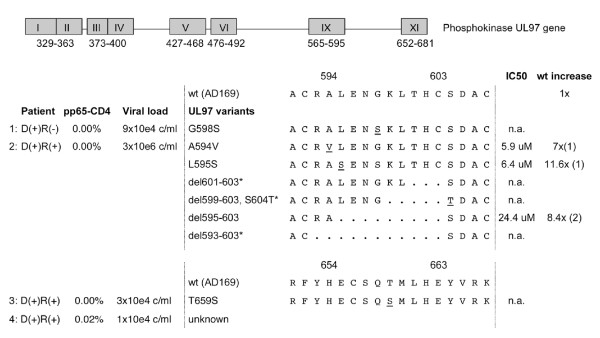
**CMV UL97 mutant variants**. Boxes indicate mutation clusters in CMV UL97 phosphokinase gene. Characters indicate amino acids; changed amino acids are underlined. Ganciclovir (GCV)-inhibitory concentrations 50% (IC50) associated with mutations are listed as μM and -fold increase over wild type AD 169 (1) or Towne (2) strain.

One of 25 D(+)R(-) KT patients (4%) developed persistent CMV replication with CMV syndrome, colitis and clinical resistance to GCV treatment, which was confirmed virologically by the identification of the CMV UL97 mutation G598S (Figure 6). In R(+)-patients, clinical resistance to GCV-treatment was identified in 3 out of 48 (6.25%) cases. CMV-specific T-cells IFNγ responses were < 0.03% for CMV-pp65 antigens (p = 0.041, two-sided Fisher-Test). Two of these R(+)-patients developed CMV syndrome with thrombocytopenia and CMV colitis, respectively. In the latter patient, we identified 6 coexisting variants, including three novel in-frame deletions in addition to a previously reported UL97 del1595-1603 known to increase the IC50 by 8.4-fold to 22.4 μM GCV (Figure 6).

## Discussion

Clinical studies have linked the lack of CMV-specific T-cells in SOT recipients to an increased risk of CMV replication and subsequent disease being most striking for CMV D(+)R(-) SOT patients [13]. CMV R(+) patients also develop CMV complications [16,17,23] albeit at lower frequencies than CMV D(+)R(-) patients. In this study, we present evidence that CMV viremia in R(+) KT-patients is associated with lower CMV-specific T-cell frequencies in PBMC. Among CD8+T-cells, this association was best captured by pp72 and pp65-specific responses, whereas in the CD4+T-cell subset, CMV-lysate and CMV-pp65 specific responses appeared to resolve this difference more effectively. ROC analysis indicated that pp65-specific CD4+ T-cell responses showed the highest AUC and seemed to provide a better trade-off between sensitivity and specificity than the other CMV-specific T-cell responses. Previous studies suggested that CMV-specific CD4+T-cell responses reflect long-term CMV surveillance, whereas CD8+T-cell responses are operative in short-term clearance of CMV replicating cells. Consistent with this hypothesis, Radha et al [22] found that D(+)R(-) KT patients who developed CMV-specific CD8+ T-cell responses cleared CMV replication more rapidly than patients without this response. In the latter patients, administration of CMV-hyperimmunoglobulin helped to clear CMV viremia suggesting that humoral immunity contributed to CMV control in D(+)(R-) patients. The antibody-enhanced clearing could involve neutralization, but also opsonization enhancing MHC-II presentation and priming of CMV-specific CD4+T-cells. Clearly, CMV-hyperimmunoglobulin deserves further study in cases with persisting CMV replication, with low specific CD4+T-cells [22] and GCV-resistance [12].

Bunde et al [17] reported that higher pp72-specific CD8+ T-cell frequencies were associated with a decreased risk of CMV disease, but not CMV replication, during the first month after heart or lung transplantation (AUC 0.719, specificity 100%, sensitivity 50%, p = 0.012). No association with pp65-specific responses was observed, which seems discrepant to our results in R(+) KT patients. It should be pointed out that heart or lung patients are generally more immunosuppressed and that all patients in the latter study received induction with antithymocyte globulin plus steroid pulses. We suspect that thereby, the kinetics of acute CMV replication were accelerated [5] such that mounting of a pp72-specific response was not rapid enough to protect from replication, but still affected progression to disease. This notion is also in line with the early pp72-specific CD8+T-cell response in 4 other cases of primary CMV replication [21]. Clearly, further studies are required to elucidate the partly divergent results and dynamics of CMV antigen-specific responses in different risk and transplant patients [19].

An important caveat of defining the risk of CMV replication through CMV-specific immune effectors resides in the dynamic aspect of the virus – host balance which is exquisitely sensitive to changes of the net state of immunosuppression. In particular, it cannot be decided whether the association of lower numbers of CMV-specific T-cells is the cause or the consequence of CMV replication. Clearly, positive CMV PCR results in blood identify patients at higher risk for CMV-associated complications where CMV replication dynamics may be helpful to predict the further course [27-29]. Negative CMV PCR results, however, are difficult to interpret with regard to future risk. In this study, we observed that pp65-specific CD4+T-cell frequencies above a threshold of 0.03% were predictive of a CMV viremia-free time for the following 8 weeks. This threshold yielded a specificity of 100% and sensitivity of 47%. The high specificity and the positive predictive value of ≥ 95% suggests clinically value because a test above this threshold would not put patients at risk for CMV replication or recurrence. The low negative predictive value of ≤ 40% appears to flag more patients for CMV surveillance than needed, but avoiding undiagnosed replication and progression to disease. Clearly, increasing calcineurin inhibitor levels, anti-rejection treatments particularly with antilymphocyte agents and steroid pulses are known to perturb antiviral immune control with lowered CMV-specific T-cell responses [21,23] and subsequent CMV replication [30,31]. With this limitation in mind, CMV pp65-specific CD4+ T-cells might serve as a dynamic marker of protection for patients on stable immunosuppression complementing CMV load diagnostics in centers using a preemptive strategy [21-23].

Our systematic comparison of CMV-lysate, -pp65 and -pp72 responses indicated that all three antigen preparations provided by and large interchangeable results, but we detected quantitative and qualitative differences in the response profiles. First, CMV-lysate responses were higher than the responses elicited by overlapping 15-mer peptide pools covering pp65 or pp72. Since control responses to non-infected fibroblast lysate were generally low and always subtracted from the individual CMV-lysate responses, these quantitative differences may reflect the wider range of CMV antigens contained in CMV-lysate preparations compared to peptide pools restricted to pp65 or pp72. Second, CMV-lysate favored CD4+ T-cell responses, whereas CMV-pp72 peptides favored CD8+ T-cell responses. The stronger CD4+ response to CMV-lysate has been reported previously [16,19,32] and may result from uptake, processing and preferential presentation of larger number of CMV-lysate antigens in an MHC-class II context. Sylwester et al [26] documented differences in immunogenicity among the 213 CMV encoded open reading frames where CMV pp65 and pp72 clearly represented dominant antigens. Compared to CMV-lysate, 15 mer peptide pools may be more eligible for direct binding to MHC-class II and also for processing to 8- to 10 mer peptides when binding to MHC-class I molecules. However, the preferential CD8+ over CD4+ response of pp72 peptide pools compared to pp65 cannot be easily reconciled. Possibly, additional sequence-encoded differences in epitopes, binding, and/or processing between pp65 and pp72 15 mer peptides must be operating as well. These inherent differences of pp72 inducing weak CD4 and strong CD8 T-cells may also explain the only borderline resolution observed between our non-immunosuppressed HD and KT patients.

Lack of CMV-specific immunity in D(+)R(-) KT- and pancreas-KT patients has been associated with an increased risk of GCV-resistance CMV replication [33]. In our study, clinical GCV-resistance as defined by Preiksaitis et al [12] occurred in 1/25 (4%) D(+)R(-) KT patients, at a rate comparable to other studies [14,15]. By contrast, the frequency of GCV-resistance in our R(+) KT patients was with 3/48 (6.25%) higher then reported previously [14,15]. Mutations in the CMV UL97 phosphotransferase have rarely been described in R(+) SOT patients to date, and, to the best of our knowledge, were not reported in KT patients [15,34]. Among UL97 mutations, A594V and L595S was identified in 30% and 13.3% of reported cases, respectively, whereas T569I and G598S mutations are less frequent [15,34-37]. Interestingly, we identified additionally 6 coexisting mutants in a single patient including three novel in-frame deletions suggesting the dynamic emergence of genotypic resistance selection during persistent CMV replication (Figure 6). Radha et al reported that persisting CMV replication in KT patients with low CMV-specific T-cell responses is not necessarily due to GCV-resistance [22]. Our study adds that low CMV-specific T-cell activity may be a first step towards selecting antiviral resistance, particularly during episodes of sub-optimally dosed antivirals in outpatients with changing renal function.

The limitations of our study are the cross-sectional approach and, although being one of the largest studies, the still relatively small sample size of KT patients. We examined CMV cellular immunity in a preemptive setting, where CMV replication represents only a surrogate marker of the risk of CMV disease. CMV replication has been used as outcome marker in other studies since CMV-disease has become rare with appropriate antiviral treatment [21,38,39]. Moreover, CMV replication without overt disease may still cause indirect effects and recently has been associated with impaired long-term graft and vasculopathy [8-10]. Nevertheless, 1 of 25 D(+)R(-) and 2 of 48 R(+) of our patients developed CMV disease (1 CMV syndrome, 2 CMV colitis). Finally, variations associated with the laboratory techniques may preclude the direct adoption of our threshold values by other institutions without further standardization, since the frequencies of measured CMV-specific T-cells may vary due to difference in stimulation protocols and degree of immunosuppression in different patients posttransplant. However, our data were obtained from studying KT patients in a clinical routine situation and therefore warrant larger, preferably prospective validation.

## Conclusion

Monitoring CMV-specific T-cell frequencies may help to identify R(+) KT patients at risk for CMV replication and possibly antiviral resistance. Provided further corroborating evidence, CMV-pp65 CD4+ responses above 0.03% in PBMCs of KT patients under stable immunosuppression are associated with lower risk of concurrent and future CMV replication during the following 8 weeks. Together with CMV blood loads, CMV-specific cellular immune responses may help to capture the dynamic interplay of the virus – host balance in transplant patients and optimize decisions concerning the dosing and duration of immunosuppressive and antiviral drugs.

## Competing interests

The authors declare that they have no competing interests.

## Authors' contributions

AE helped to recruit patients, carried out immunoassays, analyzed data and wrote the manuscript, SB carried out immunassays, AD conducted the CMV genotypic resistance testing, advised on the manuscript and generated figures, IB, CJ, SS and JS recruited patients, provided samples and advised on the study, US and MS advised on immunoassays and the manuscript, HH designed the study, analyzed the data and wrote the manuscript. All authors read and approved the final manuscript.
